# Seasonal and Diurnal Variations of Atmospheric Non-Methane Hydrocarbons in Guangzhou, China

**DOI:** 10.3390/ijerph9051859

**Published:** 2012-05-11

**Authors:** Longfeng Li, Xinming Wang

**Affiliations:** 1 School of Chemistry and Materials Science, Huaibei Normal University, Huaibei 235000, China; Email: lilongfeng@chnu.edu.cn; 2 State Key Laboratory of Organic Geochemistry, Guangzhou Institute of Geochemistry, Chinese Academy of Sciences, Guangzhou 510640, China

**Keywords:** non-methane hydrocarbons, seasonal and diurnal variations, photochemical reactivity, ozone formation

## Abstract

In recent decades, high ambient ozone concentrations have become one of the major regional air quality issues in the Pearl River Delta (PRD) region. Non-methane hydrocarbons (NMHCs), as key precursors of ozone, were found to be the limiting factor in photochemical ozone formation for large areas in the PRD. For source apportioning of NMHCs as well as ozone pollution control strategies, it is necessary to obtain typical seasonal and diurnal patterns of NMHCs with a large pool of field data. To date, few studies have focused on seasonal and diurnal variations of NMHCs in urban areas of Guangzhou. This study explored the seasonal variations of most hydrocarbons concentrations with autumn maximum and spring minimum in Guangzhou. The diurnal variations of most anthropogenic NMHCs typically showed two-peak pattern with one at 8:00 in the morning and another at 20:00 in the evening, both corresponding to traffic rush hours in Guangzhou, whereas isoprene displayed a different bimodal diurnal curve. Propene, ethene, *m*, *p*-xylene and toluene were the four largest contributors to ozone formation in Guangzhou, based on the evaluation of individual NMHCs’ photochemical reactivity. Therefore, an effective strategy for controlling ozone pollution may be achieved by the reduction of vehicle emissions in Guangzhou.

## 1. Introduction

In recent decades, high ambient ozone (O_3_) concentrations have become one of the major regional air quality issues in the Pearl River Delta (PRD) region, and from 1994 to 2007 the O_3_ levels in the PRD region had been increasing at a rate of 0.58 ppbv·year^−1^ [1]. The ground-level O_3_ is produced by photochemical reactions of non-methane hydrocarbons (NMHCs) with nitrogen oxides in the presence of sunlight. NMHCs, as key precursors of ozone, are found to be the limiting factor in photochemical ozone formation for large areas of the PRD [2,3]. Thus, an effective strategy for controlling O_3_ pollution in urban areas can be obtained by controlling the emission of NMHCs. For source apportionment of NMHCs as well as ozone control strategies, it is necessary to obtain typical seasonal and diurnal patterns of NMHCs in the PRD.

Several studies [4,5,6,7] have paid attention to the seasonal and diurnal variations of NMHCs in Hong Kong, located in the PRD. However, the seasonal and diurnal variations of NMHCs in other cities of the PRD region have not been investigated adequately. Recently, Tang *et al.* [8] reported that diurnal variations of NMHCs showed a two-peak pattern in Guangzhou in the PRD. Wang *et al.* [9] found that there were distinct different diurnal patterns of NMHCs between Guangzhou and Xinken of the PRD, whereby peak concentrations of NMHCs were usually seen at the traffic rush hour in Guangzhou, while the maxima occurred between late night and early morning in Xinken.

Although the two studies mentioned above have investigated the diurnal variations of NMHCs in Guangzhou, there is still a lack of studies on the seasonal and diurnal variations of NMHCs. Moreover, Guangzhou is considered to be the major source area of volatile organic compounds (VOCs) in the PRD. Therefore, there is an urgent need to better understand the characteristics of temporal variation of NMHCs in Guangzhou for source apportionment of NMHCs and effective control of photochemical O_3_ pollution in Guangzhou and even the larger areas in the PRD.

In this study, NMHC measurements were carried out at an urban site in Guangzhou from March to December 2005. The ambient concentrations of NMHCs in Guangzhou were examined and compared with other cities. The seasonal and diurnal variations of NMHCs concentrations, as well as the photochemical reactivity of individual NMHCs were characterized. To our knowledge, our research is the first to systemically investigate the comprehensive characteristic of NMHCs in the urban atmosphere of Guangzhou. Therefore, this study is to be a very significant work to formulate ozone pollution control strategies and help the local government implement air quality standards.

## 2. Experimental

### 2.1. Site Description

The sampling site is located at the Guangzhou Institute of Geochemistry, Chinese Academy of Sciences in the Tianhe District of Guangzhou, South China. The site is surrounded with scattered schools, commercial shops and residential buildings. In addition, the east, south and west of the site have separately several large factories such as petrochemical plants and Guangzhou Honda Co., Ltd, the Guangyuan Expressway and Huanan Expressway Trunk. Therefore, the site is expected to be adequate for representing the mixture of various VOCs emission sources. Air samples were collected on the roof of a 4-floor building (about 12 m above ground). 

### 2.2. Sample Collection

Pre-vacuated 2 L stainless steel electro polished canisters provided by University of California, Irvine (UCI) were used to collect air samples. Prior to sampling, all canisters were cleaned to –50 millitorr using a vacuum cleaner (Entech 3100A), and then each canister was joined to a flow controlling device to obtain one-hour integrated air sample (2 L). The field sampling was carried out on 6–7 March, 2–4 August, 1, 14 and 28 September, 12 October, 9 November and 9–12 December 2005, respectively. An air sample was collected at one or two-hour intervals from 6 a.m. till 22 p.m. during sampling days to explore the diurnal variations of the hydrocarbons. A total of 145 air samples were collected during the measurement periods. 

### 2.3. Analytical Procedure

The analysis of the canister samples was performed in VOCs laboratory of the State Key Laboratory of Organic Geochemistry, Guangzhou Institute of Geochemistry. A cryogenic pre-concentrator (Entech Instrument 7100A) together with a gas chromatograph (Hewlett Packard, 6890) equipped with a nonpolar capillary column (HP-1, 60 m × 0.32 mm × 1.0 μm) and a quadrupole mass spectrometer detector (MSD, Hewlett Packard 5973) were used to detect VOCs. 

First, an ambient air sample was pumped into the pre-concentrator with a 3-stage cryotraps (Module 1~3), initially trapped cryogenically by liquid nitrogen at −150 °C on glass beads of Module 1, and then desorbed at 10 °C to remove most of the liquid H_2_O. Secondly, the second cryotrap was cooled to −40 °C, allowing trapping of VOCs and leaving CO_2_. Subsequently, VOCs were backflushed from Module 2 at 180 °C and focused in the Module 3 trap at −150 °C again. Finally, after concentrated by a 3-stage cryotraps, VOCs along with helium entered into a gas chromatograph column. Column was initially held at −50 °C for 3 min, then was raised up to 10 °C at the rate of 15 °C·min^–1^, later to 250 °C at 5 °C·min^−1^ and was remained for 10 min. Thus, VOCs were separated on capillary column, and subsequently identified by MSD in Selected Ion Mode.

At least three samples were withdrawn from each canister for the reduction of analytical error and the quality check of the analysis. Standard gas containing 108 components (supplied by UCI) was used for the identification 79 different hydrocarbons and the target 59 C_2_-C_11_ NMHCs were quantified by multipoint calibration. The detection limit for C_2_-C_11_ NMHCs was 3 pptv. The measurement accuracy was 5–10% for NMHCs. The measurement precision ranged 0.5–15% for NMHCs. The response of the instrument to NMHCs was calibrated after every eight samples using standard runs of a calibration gas with ambient concentrations.

### 2.4. Meteorological Condition

**Figure 1:**
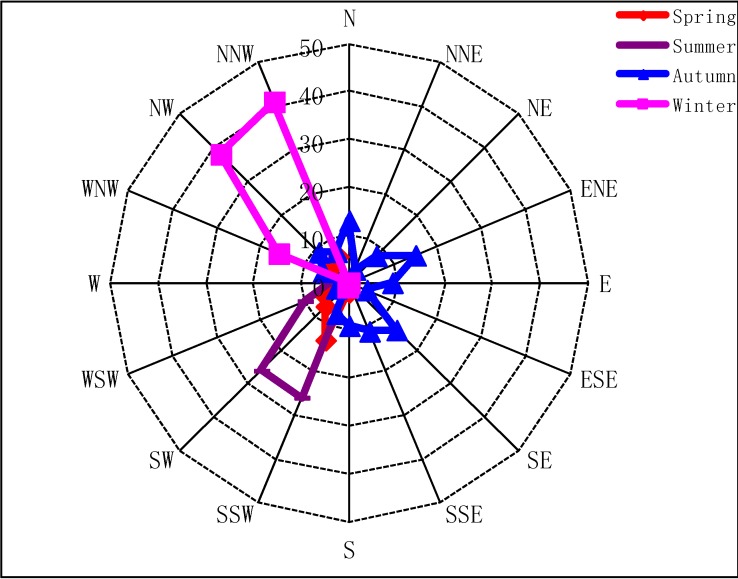
Wind frequency distribution in four seasons.

**Figure 2:**
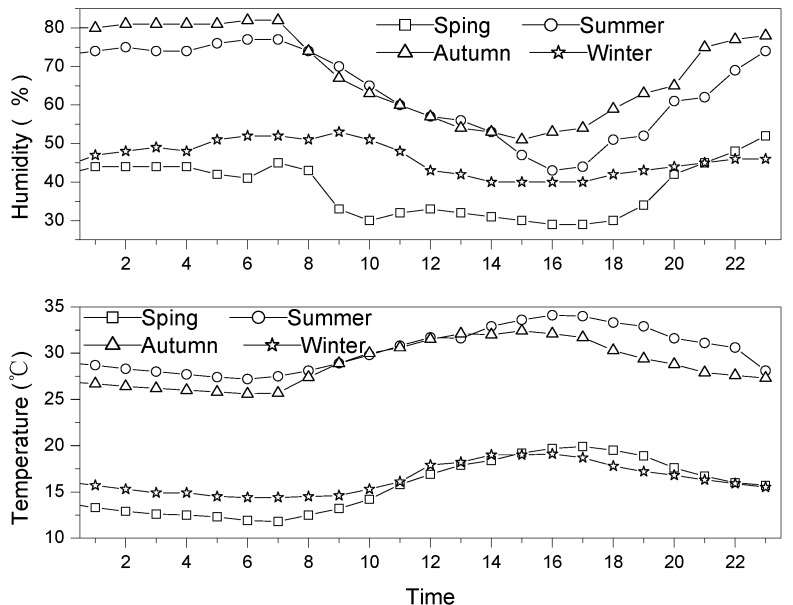
The mean temperature and humidity in four seasons.

Seasons in Guangzhou are divided into spring (March, April and May), summer (June, July and August), autumn (September, October and November) and winter (December, January and February of the following year). Figure 1 shows the wind directions during the sampling periods in the four seasons. It can be seen that southwest winds (SSW, SW and WSW) are dominant in summer, accounting for 86%, and they also account for 55% in the spring, while northwest winds (WNW, NW, and NNW) dominate the winter, and southeast, northeast and northwest winds control the autumn, accounting for 24%, 23% and 19%, respectively. The daily average temperature and the relative humidity during the sampling periods are shown in Figure 2.

## 3. Results and Discussion

### 3.1. General Characteristics of NMHCs in Guangzhou

The 59 NMHCs in urban atmosphere of Guangzhou were quantified. These 59 species including 27 alkanes, 12 alkenes, one alkynes(ethyne), 16 aromatics and three biogenic hydrocarbons (isoprene, α-pinene, and β-pinene) added up to 47.3 ppbv, in which alkanes, alkenes, alkynes, aromatics and biogenic hydrocarbons accounted for 49, 14, 12, 23 and 2% of the total NMHC concentration, respectively. The average mixing ratios and standard deviations of selected hydrocarbons in the ambient air samples are presented in Table 1. The species listed in Table 1 are abundant and account for about 87% of the total NMHC concentration in the urban atmosphere of Guangzhou. The most abundant hydrocarbon in the urban atmosphere of Guangzhou is ethyne, followed by propane and toluene (Table 1). Previous studies [8,10,11] also observed that ethyne, propane and toluene were the three most abundant species in the urban area of Guangzhou, but with a different ranking order.

**Table 1:** Mixing ratios of abundant hydrocarbons at an urban site in Guangzhou (units: ppbv).

Hydrocarbons	Mixing ratios
Ethyne	5.46 ± 4.03
Propane	4.49 ± 3.69
Toluene	4.19 ± 4.94
Ethane	3.15 ± 2.89
Ethene	2.79 ± 1.45
*n*-Butane	2.54 ± 1.80
Propene	2.26 ± 2.10
*n*-Hexane	2.11 ± 3.39
*i*-Pentane	1.81 ± 1.70
Benzene	1.73 ± 1.26
*m/p*-Xylene	1.65 ± 2.74
*i*-Butane	1.35 ± 1.02
*n*-Pentane	1.18 ± 0.97
Ethylbenzene	1.03 ± 1.49
*n*-Octane	0.79 ± 1.10
3-Methylhexane	0.72 ± 0.92
2-Methylpentane	0.70 ± 0.68
Isoprene	0.66 ± 0.90
*o*-Xylene	0.64 ± 1.03
3-Methylpentane	0.62 ± 0.58
*n*-Heptane	0.56 ± 0.75
2,3-dimethylbutane	0.52 ± 0.50
Alkanes	23.28 ± 1.09
Alkenes	6.83 ± 0.93
Alkynes	5.46 ± 4.03
Aromatics	10.80 ± 1.09
Biogenics	0.88 ± 0.33
∑NMHCs	47.26 ± 1.19

In order to better understand the general characteristics of ambient level and various source emissions of NMHCs in Guangzhou, we compare the NMHC mixing ratios of Guangzhou with other cities. A comparison of some NMHC mixing ratios in various cities is illustrated in Table 2. These NMHCs are chosen for comparison since they are usually the most abundant hydrocarbons and the markers of special emission sources in the cities. From Table 2, we can find that the mixing ratios of NMHCs in Guangzhou fall within the ranges of hydrocarbons measured at the 43 Chinese cities, although propane, propene and ethyne levels in Guangzhou are higher than in other cities. The presence of propane in the urban area of Guangzhou can be attributed to leakage from LPG-fueled vehicles [8]. Propene and ethyne are mainly emitted by vehicular combustion in urban environments [12]. Therefore, the higher levels of propane, propene and ethyne in Guangzhou in comparison with other cities indicate a higher level of vehicular emissions and lower vehicular emission control. 

**Table 2:** Comparison of selected NMHCs in Guangzhou with other cities (units: ppbv).

Hydrocarbon	Guangzhou ^a^, China	Beijing ^b^, China	Honkong ^c^, China	Dongguan ^d^, China	Shanghai ^e^, China	Kaohsiung ^f^, Taiwan	43 Cities China ^g^
Ethane	3.15	3.75	1.83	1.60	1.10	4.5	3.7–17.0
Propane	4.49	3.59	1.60	2.46	4.25	3.1	1.5–20.8
*i*-Butane	1.35	2.31	0.90	1.07	0.97	0.7	0.4–4.6
*n*-Butane	2.54	2.75	1.46	1.89	1.80	2.3	0.6–18.8
*i*-Pentane	1.81	4.11	0.52	1.42	1.98	3.8	0.3–18.8
*n*-Pentane	1.18	1.7	0.25	0.70	1.40	1.3	0.2–7.7
Ethene	2.79	4.59	1.47	3.07		7.5	2.1–34.8
Propene	2.26	1.16	0.32	0.53	0.93	2.2	0.2–8.2
Ethyne	5.46	5.41	1.95	4.27	3.58	4.9	2.9–58.3
Benzene	1.73	1.76	0.42	1.26	1.67	1.1	0.7–10.4
Toluene	4.19	3.03	2.77	6.13	4.69	8.2	0.4–11.2
Ethylbenzene	1.03	0.98	0.40	1.06	1.51	0.7	0.1–2.7
*m,p*-Xylene	1.65	2.04	0.70	1.48	1.31	1.2	0.4–15.3
Isoprene	0.66	0.41	0.18	0.68	0.03	0.6	0.04–1.7

^a ^This study; ^b ^Song *et al.* 2007 [13]; ^c ^Guo *et al.* 2007 [4]; ^d ^Barletta *et al.* 2008 [10]; ^e ^Ran *et al.* 2009 [14]; ^f ^Chang *et al.* 2005 [15]; ^g ^Barletta *et al.* 2005 [12].

Toluene is both the most abundant aromatic hydrocarbon in all cities compared and the top most abundant hydrocarbon in Hong Kong, Dongguan, Shanghai and Kaohsiung as well. However, toluene is the third most abundant hydrocarbon in Guangzhou, and the ambient level of toluene in Guangzhou is lower than that of Kaohsiung, Dongguan and Shanghai, respectively (Table 2). Toluene came directly from vehicular emissions and gasoline evaporation in Guangzhou, while in Dongguan toluene was released by industrial sources [10]. In Kaohsiung, the higher level of toluene was attributed to higher mobile vehicles emissions as well as vaporization of organic solvents used in the petroleum industries [15], whereas toluene was primarily resulted from paint solvent usage in Shanghai [16]. In conclusion, various emission sources contributed to toluene levels in different cities. The high levels of toluene in Kaohsiung, Dongguan and Shanghai indicate the strong industrial emissions in these cities. 

Isoprene was primarily emitted from biogenic sources [17], but isoprene levels were also enhanced by vehicular emissions in urban areas [18,19,20]. In Table 2, Guangzhou has a comparable isoprene level with Dongguan and Kaohsiung, which is more than Beijing, Hong Kong and Shanghai, indicating the higher emissions strength of the sources in Guangzhou, Dongguan and Kaohsiung.

### 3.2. Seasonal Variations

**Figure 3:**
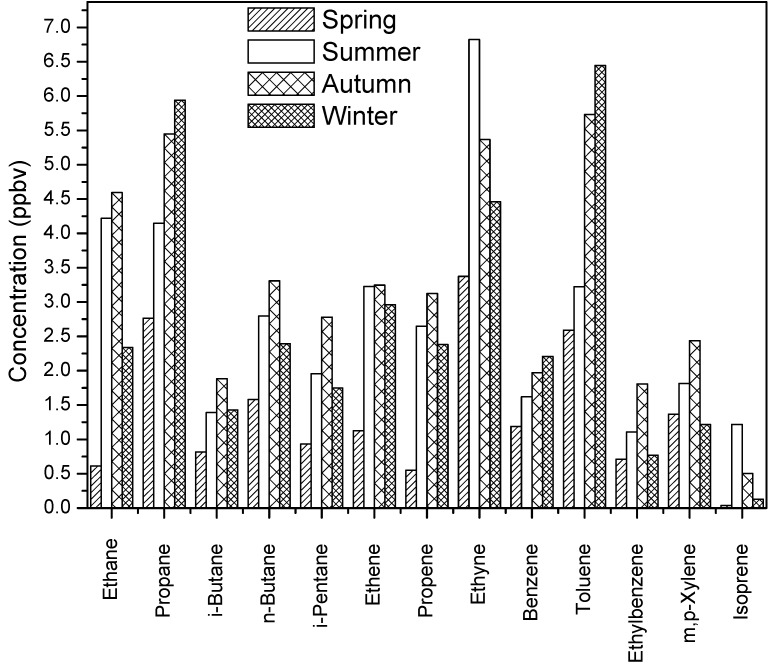
Seasonal variations of selected NMHCs in Guangzhou.

The seasonal variations of selected NMHCs in Guangzhou are illustrated in Figure 3. Ethane, *i/n*-butane, *i*-pentane, ethene, propene, ethylbenzene and *m,p*-xylene show autumn maximum concentrations while propane and toluene exhibit winter maxima. Besides, ethyne and isoprene have summer maximum concentrations. On the other hand, all selected NMHC concentrations exhibit spring minima. The seasonal variations of NMHCs in the troposphere were generally affected by various factors such as source emission strength variations, meteorological conditions and seasonal variability of OH radicals. Since OH radicals exhibited the highest concentration in summer and the lowest in winter, and the reaction with OH radicals was the major sink of NMHCs [21], the NMHC concentrations generally showed winter maxima and summer minima in many urban areas [22,23,24]. However, the seasonal fluctuations of the NMHCs concentration in Guangzhou are different from other urban areas. That is, most of the hydrocarbons show autumn maxima and spring minima concentrations. The unique seasonal patterns of the NMHCs in Guangzhou were likely dominated by meteorological factors such as the monsoon winds, since the seasonal change of monsoon winds plays a key role in controlling seasonal variation of pollutants in the PRD region [25]. 

Guangzhou enjoys a typical subtropical climate under a strong influence of the Asian monsoon. Northerly and northeasterly winds from Mainland China prevail during the autumn-winter season while southerly and southwesterly winds from the South China Sea are predominant in the summer-spring season. The continental outflows in autumn-winter enrich much more air pollutants than the marine air masses in spring-summer, as a result, the enhanced pollutants concentrations appear in autumn—winter, while the lowest concentrations occur in spring-summer. In addition, a hazy-weather phenomenon was very common in the PRD region in autumn and the air was relatively dirty under such weather conditions [26]. Consequently, it is not surprising that most of the NMHCs have autumn maximum concentrations in Guangzhou. A similar result with autumn maximum concentrations of benzene, toluene, ethylbenzene and xylenes in Guangzhou was reported by Wang *et al.* [27].

Ethyne concentration in the four seasons shows a summer maximum. This can be explained by the strong source emissions and *in situ* prevailing winds in summer. The predominant source of ethyne was traffic emissions in urban areas, and traffic emissions combined with weather conditions remarkably contributed to the ethyne concentration. As mentioned in Section 2.1, the Guangyuan Expressway and Huanan Expressway were separately located to the south and west of the sampling site. If south, west or southwest winds were prevailing during the sampling periods, the two expressways greatly impacted the ambient levels of ethyne. As expected, southwest winds are dominant in summer (Figure 1). Therefore, expressways have larger effects on the concentration of ethyne in summer than in other seasons, and this lead to the elevated concentration of ethyne in summer when the southwest winds are prevailing. 

**Figure 4:**
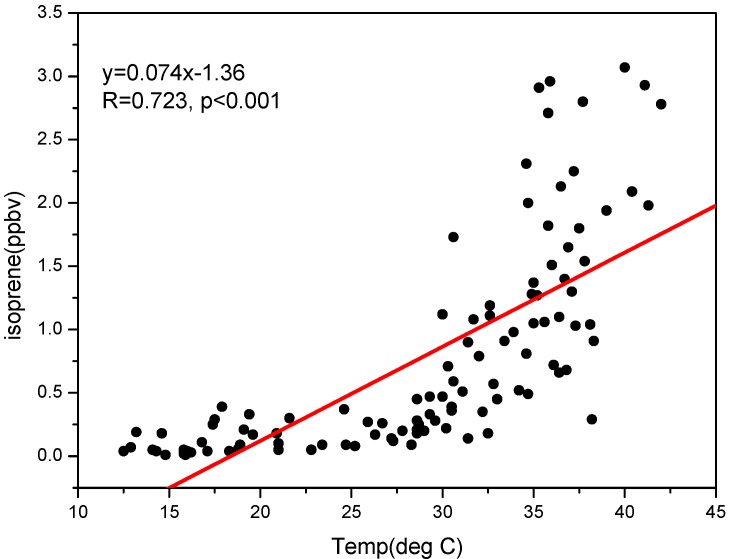
Scatter plots for isoprene *versus* temperature in Guangzhou.

Similar to the seasonal variation of ethyne concentration, isoprene also has a summer maximum concentration among the four seasons (Figure 3). Isoprene comes mainly from biogenic emissions although it was also from anthropogenic emissions in some urban areas. Since the biogenic emissions of isoprene depend on both temperature and solar radiation [28], isoprene displayed a distinct seasonal variation pattern with higher summertime concentrations and lower wintertime concentrations in rural or non-urban areas. Besides, such a seasonal behavior of isoprene has also been seen in urban areas [19], indicating the strong biogenic emissions of isoprene in urban area. In this study, isoprene also shows the maximum concentration in summer and the minimum in the cold spring (Figure 3). It probably suggests that the seasonal variation of isoprene was driven by biogenic emissions in Guangzhou. In addition, isoprene concentrations correlate well with temperature, having a correlation coefficient of R = 0.72 (Figure 4). This proved that the seasonal variation of isoprene was principally controlled by biogenic emissions in Guangzhou.

### 3.3. Diurnal Variations

**Figure 5:**
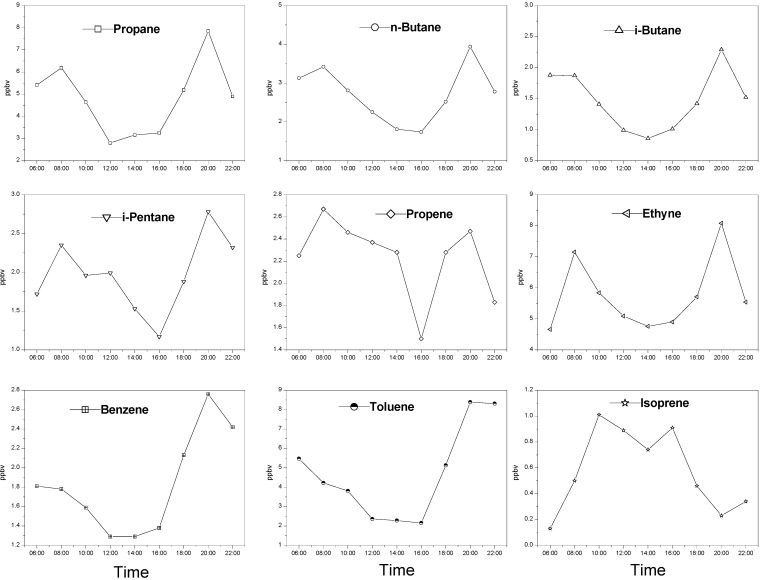
Diurnal variations of selected NMHCs in Guangzhou.

The average diurnal variations of selected NMHCs in Guangzhou are shown in Figure 5. The diurnal variations for propane, *n*-butane, *i*-butane, *i*-pentane, propene, benzene and ethyne exhibit a generally similar shape with the first concentration peak in the morning (8:00 a.m.) and the second one in the afternoon (20:00 p.m.), both corresponding to traffic rush hours in Guangzhou. The two peak patterns indicate that the predominant sources of these hydrocarbons are traffic emissions.

The toluene time series shows higher early morning and nighttime concentrations and lower levels from 12:00–16:00 (Figure 5). In detail, the high concentration of toluene appears in the early morning (6:00 a.m.) before the morning traffic rush hours. Later, the concentration of toluene gradually decreases to the lowest at noon and stays at a low value from 12:00 till 16:00 p.m., and then increases gradually to the another high value in the afternoon rush hours within a day cycle (Figure 5). The diurnal variation of toluene concentration can be explained as follows: nocturnal inversion near the surface developed by the rapid radiation of heat from the ground at night and early morning can give rise to the higher early morning concentration of toluene. Afterwards, as the sunlight continues to heat up the surface, the inversion top grows and finally breaks up around noon, which allows clean air to dilute toluene to its minimum level in a diurnal cycle. Furthermore, intense photochemical reactions with OH also contribute to the lower levels of toluene around noon. Finally, the strong emission of vehicle-related sources, together with the decreasing in the mixing layer height is responsible for the maximum concentration of toluene arising during the afternoon rush hour. 

The diurnal fluctuation of isoprene concentration shows a bimodal pattern as well. That is, isoprene has one peak concentration at 10:00 a.m. and another at 16:00 p.m. in a diurnal cycle (Figure 5). The diurnal variation of isoprene was apparently different from those of other NMHCs emitted by anthropogenic sources, and was likely driven by the local biogenic emissions. As discussed previously, the better correlations between isoprene and temperature suggest that isoprene comes mainly from biogenic emissions. Since these biogenic emissions depend on the solar radiation and temperature, the isoprene concentration goes up steadily from 6:00 to 10:00 until a peak value at 10:00 in the morning when the sun rises and the temperature increases (Figure 2). Then, the high concentration of isoprene gradually declines between 10:00 a.m. and 14:00 p.m. since photochemical reactions with OH become predominant under the intensive solar radiation. Also, at noontime, the early afternoon maximum in the intensity of the vertical mixing of the atmosphere may contribute to the decrease in the isoprene concentration. Subsequently, the concentration of isoprene increases after 14:00 and reaches another peak value at 16:00 in the afternoon within a diurnal cycle. It likely depends on the elevated temperature at 16:00 in the afternoon within a diurnal cycle (Figure 2). Finally, the reduced solar radiation and the downward temperature are responsible for the decreasing concentration of isoprene after 16:00 in the afternoon. 

### 3.4. The Photochemical Reactivity of NMHCs

As mentioned in the previous sections, the photochemical reactions with OH radicals are an important factor controlling the seasonal and diurnal variations of NMHCs. Moreover, photochemical oxidation of NMHCs results in ozone formation in the presence of NO_x_ and sunlight. However, different hydrocarbons react at different rates and exhibit differences in reactivity with respect to ozone formation. To assess individual hydrocarbons’ activity related to photochemical ozone formation, a propylene-equivalent concentration equation (1) proposed by Chameides [29] was used to calculate the propylene-equivalent concentration for individual hydrocarbon. Considering the contributions of individual hydrocarbons to photochemical ozone formation, we applied an ozone formation potentials (OFP) equation (2) to estimate OFP for individual hydrocarbon. These methods were described in detail by Tang *et al.* [8] and here unnecessary details are not given:


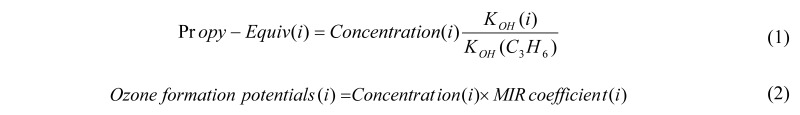


In Equation (1), Propy-Equiv(i) is a measure of species i on an OH reactivity-based scale, normalized to the reactivity of propylene, K_OH_(i) is the rate constant between species i and OH radical cited from Atkinson and Arey [30], and K_OH_(C_3_H_6_) is the rate constant between C_3_H_6_ and OH radical. In Equation (2), MIR coefficients are cited from Carter [31].

Table 3 and Table 4 lists the top 20 NMHCs ranked according to propylene-equivalent concentration and OFP value, respectively. 

**Table 3:** The top 20 NMHCs ranked according to propylene-equivalent concentration.

Rank	Species	Rate constant with OH	Propylene-equivalent concentration(ppbC)	%
1	Isoprene	100	12.50	16.0%
2	*m,p*-Xylene	18.7	9.41	12.1%
3	Propene	26.3	6.77	8.7%
4	Toluene	5.63	6.27	8.1%
5	α-Pinene	52.3	3.45	4.4%
6	*cis*-2-Pentene	65	2.83	3.6%
7	*trans*-2-Butene	64	2.71	3.5%
8	*o*-Xylene	13.6	2.63	3.4%
9	*n*-Hexane	5.2	2.51	3.2%
10	Ethylbenzene	7	2.19	2.8%
11	1-Butene	31.4	2.06	2.6%
12	*n*-Octane	8.11	1.95	2.5%
13	Ethene	8.52	1.80	2.3%
14	2-Methyl-1-butene	61	1.79	2.3%
15	*trans*-2-Pentene	67	1.70	2.2%
16	*cis*-2-Butene	56.4	1.58	2.0%
17	1-Pentene	31.4	1.35	1.7%
18	*i*-Pentane	3.6	1.24	1.6%
19	2-Methyl-2-butene	86.9	1.13	1.5%
20	*n*-Heptane	6.76	1.01	1.3%

Note: Rate constant with OH are cited from Atkinson & Arey [30].

**Table 4:** The top 20 NMHCs ranked according to ozone formation potential.

Rank	Species	MIR value	Conc (ppbv).	(%)
1	Propene	9.4	21.22	17.3%
2	Ethene	7.4	20.61	16.8%
3	*m,p*-Xylene	7.4	12.24	10.0%
4	Toluene	2.7	11.30	9.2%
5	Isoprene	9.1	5.98	4.9%
6	o-Xylene	6.5	4.13	3.4%
7	1-Butene	8.9	3.84	3.1%
8	*trans*-2-Butene	10	2.79	2.3%
9	Ethylbenzene	2.7	2.78	2.3%
10	Ethyne	0.5	2.73	2.2%
11	1,2,4-Trimethylbenzene	8.8	2.70	2.2%
12	*n*-Butane	1.02	2.59	2.1%
13	*i*-Pentane	1.38	2.50	2.0%
14	Propane	0.48	2.15	1.8%
15	*n*-Hexane	0.98	2.07	1.7%
16	*cis*-2-Butene	10	1.84	1.5%
17	*i*-Butane	1.21	1.64	1.3%
18	1-Pentene	6.2	1.40	1.1%
19	1,2,3-Trimethylbenzene	8.9	1.37	1.1%
20	*n-*Pentane	1.04	1.23	1.0%

Note: MIR values are taken from Carter [31].

These NMHCs accounted for about 86% of the total propylene-equivalent concentration and about 87% of the total OFP at the urban site in Guangzhou. Isoprene, *m,p*-xylene, propene and toluene are the top four hydrocarbons based on propylene-equivalent concentrations, accounting for 16%, 12%, 9% and 8% of the total concentration, respectively. The rests mostly are alkenes. This result was consistent with the estimates in the PRD [8].

Propene, ethene, *m,p*-xylene and toluene are the top four contributors to the OFP at the Guangzhou urban site, which separately accounted for 17%, 17%, 10% and 9% of the total OFP. The high contributions of propene, ethene, *m*,*p*-xylene and toluene to the total OFP had also been reported by other studies [8,18]. 

The alkenes (ethene and propene, *etc.*) and the aromatics were mainly emitted from vehicular source although toluene and *m,p*-xylene were also from gasoline and solvent evaporation. Therefore, vehicles emissions can dramatically contribute to the ozone formation in Guangzhou. 

Isoprene, primarily from biogenic emissions, accounted for 16% of the total propylene-equivalent and 5% of the OFP, so biogenic emissions were also an important contributor to the ozone formation in Guangzhou.

## 4. Conclusions

The ambient atmospheric concentrations of 59 NMHCs (27 alkanes, 12 alkenes, one alkyne, 16 aromatics and three biogenic hydrocarbons) were measured at an urban site in Guangzhou during March–December 2005. Alkanes provided the largest contribution to the total NMHCs, accounting for over 49%. Alkenes, alkynes, aromatics and biogenic hydrocarbons separately accounted for 14, 12, 23 and 2% of the total NMHC concentrations. Ethyne, propane and toluene were the three top most abundant individual hydrocarbons in the urban atmosphere of Guangzhou. 

The seasonal variation trends of most anthropogenic emissions of NMHCs generally showed high concentrations in autumn and low concentrations in spring. Such seasonal behaviors were mainly attributed to the seasonal change of monsoon winds in the PRD. Isoprene revealed its highest summer concentration among the four seasons, suggesting its well-known biogenic emissions. The diurnal profiles for anthropogenic NMHC concentrations generally exhibited a first peak in the morning and a second one in the afternoon, both corresponding to traffic rush hours in Guangzhou, indicating that the predominant sources of these hydrocarbons are traffic emissions. Isoprene concentrations display a bimodal diurnal curve different from those of anthropogenic NMHCs, which was significantly affected by meteorological conditions and source emissions as well as photochemical removal.

Propene, ethene, *m,p*-xylene and toluene were the four largest contributors to the OFP, indicating that reduction of vehicles emissions could probably effectively control ozone pollution. In addition, biogenic emissions can also contribute to the ozone formation since isoprene is the fifth largest contributor to the OFP in Guangzhou.
